# Head and Neck Cancer Patients’ Quality of Life: Analysis of Three Instruments

**DOI:** 10.30476/DENTJODS.2019.77677.0

**Published:** 2020-03

**Authors:** Elâine Patrícia Alves de Araújo Gomes, Andreza Maria Fabio Aranha, Alvaro Henrique Borges, Luiz Evaristo Ricci Volpato

**Affiliations:** 1 Cuiabá Dental School, Universidade de Cuiabá, Cuiabá, MT, Brazil; 2 Postgraduate Program in Dental Science, Cuiabá Dental School, Universidade de Cuiabá, Cuiabá, MT, Brazil

**Keywords:** Adjuvant chemotherapy, Head and neck neoplasms, Inquiries and Questionnaires, Oral neoplasms, Quality of life, Radiotherapy

## Abstract

**Statement of the Problem:**

Head and neck cancer treatment has provided better cure and survival rates but the patient’s quality of life is still an issue.

**Purpose:**

To verify the correlation between the three most used instruments for evaluating the quality of life of head and neck cancer patients.

**Materials and Method:**

This cross-sectional study evaluated patients treated for head and neck cancer at the Mato Grosso Cancer Hospital, Cuiabá, MT, Brazil. The variables age, gender, cohabitation status, education, religion, smoking, ethnicity, tumor location and histological type and treatment modality were collected. The patients quality of life was assessed by the Functional Assessment of Cancer Therapy Quality of Life Measurement System (FACT-H&N), University of Washington Quality of Life Questionnaire (UW-QOL), and EORTC QLQ-C30/EORTC QLQ-H&N35 of the European Organization for Research and Treatment of Cancer.

**Results:**

The study population consisted of 33 individuals with a mean age of 63.42±11.25 years; 69.70% were males; 54.55% had no partner; 45.45% had only elementary education; 87.9% followed a religion; 84.38% were smokers and 87.50% alcoholics. Squamous cell carcinoma responded for 78.79% of the cases and palate/oropharynx and mouth floor (21.21% each) were the most affected sites. All patients underwent radiotherapy, 90.91% chemotherapy and 63.64% surgery. On the analysis of quality of life, shoulder (UW-QOL), social performance (EORTC QLQ-C30/QLQ-H&N35) and overall well-being (FACT-H&N) had the highest scores while saliva (UW-QOL), nausea and vomiting (EORTC QLQ-C30/QLQ-H&N35) and emotional well-being (FACT-H&N) had the lowest scores. A positive correlation was found between the questionnaires for the patient's overall quality of life and the domains Pain, Appearan-ce, Activity, Deglutition, Chewing, Speech, Taste, Saliva, Mood and Anxiety.

**Conclusion:**

Given the correlation between the questionnaires, the selection of the instrument for future research involving head and neck cancer patients’ quality of life should consider the specific aspects to be evaluated.

## Introduction

The term "head and neck cancer" is defined by anatomical and topographic bases to describe malignant upper aerodigestive tract tumors, including oral cavity, pharynx and larynx [ 1 ]. It is the nineth most common malignant neoplasm in the world, with high incidence, prevalence and mortality [ 2 ]. Despite cancer treatment has achieved better results in recent decades, providing a significant increase in patient survival [ 3 ], head and neck cancer patients, as well as having a life-threatening disease, have to deal with the impact of their treatment on functional and aesthetic aspects. The affected region is the anatomical site of basic functions, such as speech, swallowing, hearing and breathing, which are of vital importance for an individual, besides being related to social interaction [ 4 - 5 ]. Thus, researchers have given greater attention to the assessment of quality of life (QOL) of these patients [ 3 ].

To evaluate the quality of life of patients with cancer is important to be able to understand the impact of the disease and its treatment on the daily routine of the patient and to improve the protocol of care with more comprehensive clinical, social and rehabilitation support measures [ 6 ]. Quality of life studies in head and neck cancer patients in chemotherapeutic and radiotherapeutic treatment have evaluated the side effects of treatment and assisted in the planning of interventions to reduce both physical and psychological stress for better patient rehabilitation [ 7 - 8 ]. As patient survival has increased and as the disease and its treatment impact on their quality of life, it was observed the need to study the quality of life among these patients [ 3 ]. The assessment of quality of life enables health professionals to understand how patients experience the evolution of the disease, the impact of the disease on their life, the sequelae of the treatment and relapse of the disease, as well as the effectiveness and consequences of the treatments and of the care offered [ 6 ].

The most used instruments for quality of life analysis of patients with head and neck cancer, according to the International Conference on Quality of Life held in Virginia, USA, in October 2002, are the Functional Assessment of Cancer Therapy Quality of Life Measurement System (FACT-H&N), the University of Washington Quality of Life Questionnaire (UW-QOL) and the European Organization for Research and Treatment of Cancer Quality of Life Questionnaire Core 30- EORTC QLQ-C30 alog with the European Organization for Research and Treatment of Cancer Quality of life-Head and Neck Cancer Module (EORTC QLQ-H&N35) [ 6 ].

The FACT-H&N evaluates a health-related quality of life in patients with head and neck cancer. The questionnaire contains 38 items, 7 on physical well-being, 7 on social/family well-being, 6 on emotional well-being, 7 on functional well-being and 11 on additional and specific concerns. The scores of the answers vary from "not at all" (score 0) to "very" (score 4) and, the higher the score, the better the positivity in relation to the domain measured. The UW-QOL (Version 4) has 12 specific questions about different dimensions of quality of life (pain, appearance, activity, recreation, deglutition, chewing, speech, shoulder, taste, saliva, mood and anxiety). Each issue allows to describe how dysfunctions or limitations experienced daily by the patient. High QOL or normal function represents 100 points, while lower levels are represented by lower values [ 9 ]. It also presents a question that allows the patient to classify which domains are most important to him and also consists of three general questions about his overall and related to health quality of life. The EORTC QLQ-C30 (version 3) incorporates 5 functional scales (physical, performance, cognitive, emotional and social), 3 symptom scales (fatigue, pain, nausea and vomiting), global health status / QOL scale and 6 simple items for assessment of symptoms or additional problems (dyspnea, loss of appetite, insomnia, financial difficulties, constipation and diarrhea). The scores of the questionnaire range from 0 to 100. Regarding functional and overall health status scales, higher scores relate to better quality of life; however, for the scales of symptoms, higher scores correspond to the higher presence of this symptom and, consequently, the worse quality of life [ 10 ]. Its specific module for head and neck cancer patients (EORTC QLQ-H&N35) comprises 35 questions about symptoms and side effects of treatment, social function, body image and sexuality. It incorporates 7 symptom scales (pain, swallowing, taste and smell, speech, eating in public, social contact and sexuality) and 11 simple items. For all scales and simple items, a high score indicates poorer quality of life [ 11 ]. The data obtained corresponds to the patient's condition during the last week [ 12 ].

As the questionnaires present their particularities, this should be considered for the selection of the most appropriate instrument for each research, according to the purpose of the study. However, we did not find a study that analyzed whether there is a correlation between the three instruments most commonly used to evaluate the quality of life of patients with head and neck cancer. Thus, this study aims to analyze the correlation between the three instruments of quality of life analysis of patients with head and neck cancer and, therefore, to help researchers in the selection of the most appropriate instrument to evaluate the quality of life of their patients.

## Materials and Method

### Characteristics of the population

The study population consisted of patients who met the following criteria: they were diagnosed with cancer in the head and neck region; have been undergoing antineoplastic treatment for cancer treatment and the treatment has been completed for at least six months. These patients were recruited by convenience in the Department of Dentistry of the Mato Grosso Cancer Hospital, Cuiabá, MT, Brazil.

### Eligibility criteria

Participated in the research were men and women over 18 who underwent treatment for head and neck cancer completed at least six months previously with follow-up at the Department of Dentistry of the Hospital de Cancer de Mato Grosso; and signed the free and informed consent form.

### Ineligibility criteria

Patients who underwent antineoplastic treatment in other institutionsand patients in use of analgesics and anxiolytics.

### Questionnaires application

Initially each individual was instructed to complete an identification form containing fields related to age, sex and social habits of smoking and alcoholism.

The patient's medical records were also searched for information on the location and histological type of the tumor; antineoplastic treatment performed by the patient; total dose of radiotherapy, date of completion of treatment. In order to collect quality of life data, three validated questionnaires for the Brazilian Portuguese language[6,13] were used: (1) Functional Assessment of Cancer Therapy Quality of Life Measurement System (FACT-H&N) (Version 4.0); (2) University of Washington Quality of Life Questionnaire (UW-QOL) (Version 4); (3) European Organization for Research and Treatment of Cancer Quality of Life Questionnaire Core 30 (EORTC QLQ-C30 version 3) together with EORTC QLQ-H&N35.

Patients were invited to participate in the survey and to respond to the questionnaires always in the morning, on the pre-scheduled days for their follow-up visits. Each patient was instructed to answer the three questionnaires, always in the same order, individually, in a reserved room. The questionnaires were self-administered and only when the patient had any doubts in filling the questionnaire the researcher read the question to the patient.

The analysis of individuals' quality of life was performed according to each instrument, for FACT-H&N, it should be emphasized that the affirmations that bring negative contents to the patient have an inverse summation, that is, the less they occur to the patient, the greater the result of quality of life. The sum of each questionnaire area ranges from 0 to 28, while the total score varies from 0 to 144. The higher the value, the better the quality of life [ 6 ]. For the UW-QOL (Version 4), the total quality of life score is given by the response category with a score ranging from 0 (worst) to 100 (best), where a compound score is also calculated, which is the mean of the twelve domains. The higher the value, the better the individual's quality of life [ 6 ]. In EORTC QLQ-C30 (version 3) and EORTC QLQ-H&N35, scales and subscales are evaluated using scores ranging from 0 to 100 such that in the first two scales (Functional and Global Health Status), the higher score, the better the quality of life, whereas in the last scale (symptoms), the interpretation is in the opposite direction, the higher the symptom score, the worse the quality of life [ 10 , 14 ] (Table 1).

**Table1:** Instruments used to evaluate the quality of life of patients with head and neck cancer in this study

Instruments	FACT-H&N	UW-QOL	EORTC QLQ-C30 / EORTC QLQ-H&N35	
			EORTC QLQ-C30	EORTC QLQ-H&N35
Number of Itens	38	12	30	35
Number of Evaluated Domains	5	12	16	18
Names of domains and scales evaluated	Physical well-being, family social well-being, emotional and functional well-being. 12 specific questions about head and neck cancer: pain, saliva, voice, appearance, swallowing, communication, alcohol consumption and smoking.	Pain, appearance, activity, recreation, deglutition, chewing, speech, shoulder, taste, saliva, mood and anxiety.	Five scales of functionality: physical performance, functional performance, emotional performance, cognitive performance and social performance; 3 scales of symptoms: fatigue, pain, nausea and vomiting; 1 global health scale; 6 items of other symptoms: dyspnea, lack of appetite, insomnia, constipation and diarrhea; 1 scale of financial impact assessment	Pain, swallowing, cognitive problems, speech, eating in public, social contact, sexuality, dental problems, open mouth, dry mouth, sticky saliva, cough, malaise, consumption of analgesics, nutritional supplements, tube feeding, loss and gain of weight.
Score	0-144	0-100	0-100	

Afterwards, the correlation of the instruments was analyzed. To analyze if there was a correlation between the questionnaires, that is, the dimensions measured by the different instruments lead to the same conclusion, it was necessary to standardize the meaning of the scores. The defined pattern was the higher the score, the higher the quality of life. For example, the answers to FACT-H&N questions vary from 0 to 4. Thus, 0 will correspond to the worst possible quality of life measured by the questionnaire and 4 the best quality of life possible. UW-QOL follows the same pattern with high QOL or normal function representing 100 points, while lower levels are represented by lower values. In the EORTC QLQ-C30 questionnaire, on functional and global health status scales, higher scores relate to better quality of life; however, for the scales of symptoms, higher scores correspond to the greater presence of said symptom and, consequently, the worse quality of life. Thus, for the correlation analysis, on the EORTC QLQ-C30 symptom scale, the score was inverted when the data was transcribed to the spreadsheet, so that the highest scores were equivalent to the best QOL.

### Statistical analysis

The data of the questionnaires were manually transferred to an Excel spreadsheet (Microsoft, Albuquerque, New Mexico, United States), which served as the basis for the analysis using SPSS software 20.0 (Statistical Package for the Social Science, IBM, Chicago, United States) and SAS 9.0 (Statistical Analysis System, StatSoft, Cary, North Carolina, USA).

Initially, a descriptive analysis of the patients' variables and their treatment was performed using absolute and relative frequency. Regarding the patients' ages, the mean, standard deviation and minimum and maximum values ​​were calculated. The quality of life of the patients was then analyzed by the different instruments and their values ​​were expressed by the mean, median and standard deviation.

In order to verify the correlation between the general score obtained by each of the questionnaires (Overall quality of life (FACT-H&N), Global Health Status (UW- QOL) and General Wellness (EORTC QLQ-C30/ QLQ-H&N35) the Spearman correlation analysis was used.

In addition to the general score, Spearman's correlation analysis was also applied with the objective of verifying the presence of association of the instruments regarding the dimensions Pain, Appearance, Activity, Swallowing, Chewing, Speech, Taste, Saliva, Shoulder, Humor and Anxiety, present on at least two of the instruments. The correlation between the questionnaires was made by analyzing them two by two because it is not possible to associate the three questionnaires simultaneously. There is no simple and usual methodology for triple multivariate association; however, breaking the analysis and making the associations two to two, it is possible to come to the correct conclusions. The hypothesis tests developed in this study considered a significance of 5%, that is, the null hypothesis was rejected when p-value was less than 0.05.

The interpretation of the results is based on the correlation signal found only for the cases with statistical significance (p-value less than 0.05). Positive correlations indicate that the answers of the questions of the different questionnaires have the same orientation. Negative correlations indicate that the answers go in the opposite direction using the different instruments.

### Ethical Aspects

All the participants were clarified about the purpose and methodology of the research and gave their formal consent through the signing of the Informed Consent Term. Prior to the execution of this research, its project was submitted to the Ethics in Research Committee of the University of Cuiabá, and was approved by protocol number 1,852,857.

## Results

The total study population consisted of 33 patients with a mean age of 63.42±11.25 years; the youngest was 41 years old and the oldest 85. The majority were male (69.70%); 54.55% of the patients had no partner; 45.45% had only elementary education and 87.9% said they followed a religion. Presence of smoking was verified in 84.38% of the patients and the alcoholism in 87.50% patients. The most frequent histological type of neoplasm among patients was squamous cell carcinoma (78.79%) and the anatomic sites most frequently affected by the tumors were the palate/oropharynx and the floor of the mouth with the same percentage (21.21%). All 33 patients (100%) underwent radiotherapy, 30 (90.91%) underwent chemotherapy and 21 (63.64%) underwent adjuvant surgery for the treatment of cancer (Table 2).

**Table2:** Distribution of patients according to socio- demographic characteristics and type of treatment

Variables	N	%
**Gender**		
Male	23	69.70
Female	10	30.30
**Marital status**		
With mate	15	45.45
No companion	18	54.55
**Escolarity**		
Not Literary	2	6.06
Elementary School	15	45.45
High school	11	33.33
Higher education	5	15.15
**Religion**		
With religion	29	87.88
Without religion	4	12.12
**Smoking**		
No	5	15.63
Yes	27	84.38
**Alcoholism**		
No	4	12.50
Yes	28	87.50
**Diagnosis**		
Metastatic Carcinoma	2	6.06
Epidermoid Carcinoma	26	78.79
Mucoepidermoid Carcinoma	1	3.03
Warty Carcinoma	2	6.06
Adenocarcinoma	2	6.06
**Tumor site**		
Tongue	4	12.12
Mandible body	1	3.03
Tonsil	3	9.09
Palate / Oropharynx	7	21.21
Larynx	4	12.12
Nasopharynx	3	9.09
Floor of Mouth	7	21.21
Buccal mucosa	3	9.09
**Radiotherapy**		
No	0	0.00
Yes	33	100
**Chemotherapy**		
No	3	9.09
Yes	30	90.91
**Surgery**		
No	12	36.36
Yes	21	63.64

The quality of life of the patients assessed using the UW-QOL, EORTC QLQ-C30/QLQ-H&N35 and FACT-H&N questionnaires, as well as the score of each domain used by the different questionnaires was presented using the mean, median and standard deviation (Table 3).

**Table3:** Quality of life analysis of patients with head and neck cancer using the UW-QOL, EORTC QLQ-C30/QLQ-H&N 35 and FACT-H&N instruments expressed by mean, median and standard deviation

Variables	Mean	(n=33)	
		Median	Standard deviation
UW-QOL			
Pain	73.48	75.00	26.47
Appearance	81.06	75.00	21.68
Activity	68.94	75.00	30.64
Swallowing	50.30	30.00	27.21
Chewing	28.79	0.00	35.42
Speech	72.12	70.00	27.59
Shoulder	85.76	100	25.86
Taste	53.03	30.00	34.50
Saliva	30.30	30.00	29.42
Mood	76.52	75.00	25.72
Anxiety	79.55	100.00	32.07
Total	63.31	63.33	14.02
EORTC QLQ-C30			
Global health status/QoL	61.62	50.00	21.34
Physical functioning	68.48	66.67	25.40
Role functioning	64.65	66.67	34.04
Emotional functioning	58.08	66.67	30.86
Cognitive functioning	72.22	83.33	28.77
Social functioning	89.90	100	15.56
Fatigue	28.96	22.22	23.56
Nausea and vomiting	8.08	0.00	23.24
Pain	22.22	16.67	25.23
Dyspnoea	11.11	0.00	21.52
Sleep	36.36	33.33	40.28
Appetiite loss	28.28	0.00	39.19
Constipation	15.15	0.00	27.75
Diarrhoea	17.17	0.00	26.51
Financial difficulties	34.34	33.33	39.52
QLQ-H&N 35			
Pain	23.23	25.00	18.37
Swallowing	36.62	33.33	23.38
Senses problems	36.36	33.33	24.81
Speech problems	18.18	0.00	24.82
Trouble with social eating	33.33	25.00	26.52
Trouble with social contact	16.97	6.67	19.44
Less sexuality	57.07	66.67	37.27
Teeth	59.60	66.67	44.69
Opening mouth	26.26	0.00	39.75
Dry mouth	87.88	100	24.75
Sticky saliva	67.68	66.67	35.83
Coughing	19.19	0.00	30.08
Felt ill	15.15	0.00	27.75
Pain killers	45.45	0.00	50.56
Nutritional supplements	42.42	0.00	50.19
Feeding tube	45.45	0.00	50.56
Weight loss	42.42	0.00	50.19
Weight gain	54.55	100	50.56
FACT-H&N			
Physical well-being	15.42	16.00	4.75
Social/Family well-being	25.79	26.00	5.38
Emotional well-being	14.24	16.00	5.37
Functional well-being	25.09	26.00	5.70
FACT-G (general)	80.55	83.00	14.11
Bem-estar funcional-H&N	21.19	20.36	4.28
FACT-H&NPACP	21.97	21.00	6.13
FACT- H&N TOI (trial outcome index)	62.48	64.00	12.04
FACT-H&N	102.52	102	17.58

The quality of life measured by the UW-QOL questionnaire was (63.31±14.02). In the specific domains, the highest scores occurred in the shoulders (85.76±25.86), appearance (81.06±21.68) and anxiety (79.55±32.07). The lowest scores occurred in the items chewing (28.79±35.42) and saliva (30.30±29.42). Using the EORTC QLQ-C30/QLQ-H & N35 questionnaire the quality of life measured among the patients was (61.62±21.34). Among the domains evaluated by the questionnaire, the highest mean score was observed in social performance (89.90±15.56), followed by cognitive performance (72.22±28.77) and the lowest scores occurred in nausea and vomiting (8.08±23.24), dyspnoea (11.11±21.52), constipation (15.15±27.75) and diarrhea (17.12±26.51). 

In the specific domain, the highest values ​​were found in dry mouth (87.88±24.75) and sticky saliva with a mean of 67.68 (±35.83) and the lowest in “I felt sick” (15.15±27.75), followed by problems with social contact (16.97±19.44), speech problems (18.18±24.82) and cough (19.19±30.08). It is worth remembering that in this specific domain, the higher the score, the worse the intensity in the evaluated domain. The score found by the FACT-H&N questionnaire for patients' quality of life was 102.52 (±17.58). In the other items evaluated by the instrument, the highest values were found in FACT-G (80.55±14.11) and FACT- H&N TOI (62.48±12.04); the lowest means pre- sented were for emotional well-being (14.24±5.37) and physical well-being (15.42±4.75).

After analyzing the quality of life of the patients with each instrument, an analysis of the correlation of the instruments was performed (Table 4).

**Table4:** Correlation analysis of the UW-QOL, EORTC QLQ-C30/QLQ-H&N35 and FACT-H&N questionnaires (general score and specific domains) using Spearman's correction

Questionaires and its domains	Correlation	*p* Value
Correlation between the instruments UW-QOL e EORTC QLQ-C30/QLQ-H&N 35		
Overall Score	0.41	<.000
Pain	0.71	<.000
Appearance*	-	-
Activity	0.51	<.000
Swallowing	0.72	<.000
Chewing	0.34	0.001
Speech	0.43	<.000
Taste*	0.71	<.000
Saliva	0.74	<.000
Mood and Anxiety	0.36	0.000
Correlation between UW-QOL and FACT-H&N instruments		
Overall Score	0.28	0.005
Pain	0.63	<.000
Appearance	0.33	0.001
Activity	0.35	0.000
Swallowing	0.57	<.000
Chewing	0.61	<.000
Speech	0.44	<.000
Taste	-	-
Saliva	0.75	<.000
Mood and Anxiety	0.35	0.000
Correlation between EORTC QLQ-C30/ QLQ-H&N 35 and FACT-H&N instruments		
Overall Score	0.48	<.000
Pain	0.73	<.000
Appearance	-	-
Activity	0.55	<.000
Swallowing	0.50	<.000
Chewing	0.23	0.019
Speech	0.38	0.000
Taste	-	-
Saliva	0.73	<.000
Mood and Anxiety	0.58	<.000

* The EORTC QLQ-C30/QLQ-H&N35 does not evaluate the field Appearance and the FACT-H&N does not evaluate the field Taste

The overall score of the three questionnaires as well as the specific domains presented a statistically significant positive correlation.

## Discussion

The mean age of the patients in this study was 63.42 years; the youngest was 41 years old and the oldest was 85 years old. Those are similar to previous studies that found the sixth decade of life as the main one in the diagnosis of head and neck cancer [ 16 - 18 ]. It is also similar to the population of the other studies from different parts of Brazil, whose mean age was 63.5 [ 18 ] and 61.1 years [ 19 ]. The population of this study was predominantly male (69.7%), also agreeing with previous studies with head and neck cancer patients in Mato Grosso, Brazil and other countries [ 15 - 20 ].

Regarding the presence of companion (45.5%), schooling (45.45% studied until elementary school) and religion (87.88% followed a religion), the profile of the patients in this study is also similar to previous studies conducted in Brazil [ 18 , 21 ]. 

Regarding the habits, 84.38% and 87.50% of the individuals used or still use tobacco and alcohol respectively. All individuals who use or used alcohol have the same behavior with tobacco. Almeida *et al*. [ 22 ] found a similar prevalence of these habits. Smoking and alcoholism are considered important risk factors for the development of head and neck cancer and may interfere with the prognosis of cancer treatment [ 23 ].

Most of the patients (78.79%) presented a diagnosis of epidermoid carcinoma, which represents the most common histological type in head and neck neoplasms, especially in oral and oropharyngeal cancer [ 24 - 25 ]. It affects mainly males, older than 50 years, smokers and/ or alcoholics [ 26 ], as observed in this study.

The main tumor sites were palate/oropharynx (21.21%) and mouth floor (21.21%), followed by larynx and tongue, with 12.12% each.

The result is similar to that found by Dobrossy [ 24 ], 40% in the oral cavity, 25% in the larynx, 15% in the pharynx and the rest in the salivary and thyroid glands. Yet, in an epidemiological study conducted from 1997 to 2000 in Santos, SP, Brazil, the tongue was the most frequent site, with 51.1% followed by the mouth floor with 25.5% [ 27 ]. In the work of Perez *et al*. [ 28 ], the area most affected by cancer was the floor of the mouth, with 317 patients (57.5%), followed by the oropharynx with 140 patients (25.3%) and palate and lips with 11 cases (6.5 %) each.

Of the 33 patients, 100% underwent radiotherapy, 90.19% also underwent adjuvant chemotherapy and 63.64% underwent surgery. The antineoplastic treatment is defined on the staging of the disease [ 29 ]. In the case of head and neck tumors, radiotherapy and surgery are the most recommended treatments [ 30 ]. Radiation therapy can be used with curative or palliative intent and the application schedule depends on the total dose calculated and the radiotherapist's evaluation [ 31 ].

The study population consisted of patients treated for head and neck cancer under follow-up, recruited for convenience. Convenience samples are common in the health area because they allow the researcher to select the subjects to which they have access, assuming that they can represent a universe [ 32 ]. All the research subjects were invited to complete the three quality of life questionnaires following the guidelines for completing each instrument [ 6 , 33 ]. The procedure took place in a reserved room and a professional, always the same, was available to resolve any questions that patients might have about the questionnaires. To respond to the UW-QOL and EORTC QLQ-C30/QLQ-H&N35 questionnaires, the patients took approximately fifteen minutes and approximately ten minutes to respond the FACT-H&N. This time is twice the time reported by the creators of the instruments [ 6 ]. Two factors may have contributed to this: the age of patients, mostly elderly, and the fact that they are often not limited to just answering the questionnaires, but explaining and justifying their answers to the researcher, or even, seize the opportunity of that moment to talk.

Of the three instruments used, the only one that openly questions the individual's opinion about his QOL is UW-QOL. The instrument has twelve specific closed questions and three open questions where is observed the concern about investigating QOL before and after diagnosis/ treatment. Thus, the questionnaire considerably increases the analysis of the dynamics of QOL over time [ 34 ]. The patients considered the UW-QOL questionnaire easy to answer, although some were not satisfied with the alternatives offered since they are often specific and closed.

Analyzing the three questionnaires, it is noticed that oral cavity related topics such as pain, taste, saliva, speech and communication, swallowing and chewing are considered fundamental for the evaluation of QOL and are present in all of them. Nevertheless, some of these topics are explored more explicitly in some questionnaires than in others. In the questionnaire EORTC QLQ-30/QLQ-H&N35 the specific topics related to the oral cavity are addressed in a very explicit way, however, the UW-QOL questionnaire addresses more questions related to quality of life in relation to general health.

In this study, the lowest scores measured by UW-QOL were chewing and saliva. De Souza [ 35 ] applied the same questionnaire before, during and after the radiotherapy treatment for malignant lesions and the domains with the worst score at the end of radiotherapy were palate, saliva and swallowing. Rogers *et al*. [ 36 ] evaluated 349 patients previously submitted to surgery for cancer of the oral cavity and oropharynx using the UW-QOL questionnaire. The domains of QOL that presented statistical correlation in relation to age were pain, activity and recreation; for the gender, the most affected domains were shoulder and saliva. Li *et al*. [ 37 ] investigated the quality of life of Chinese patients with tongue cancer who underwent immediate reconstruction surgery; the patients had better performance in shoulder domains, and worse domains in appearance. Although they are different studies, a recurrence of inferior scores in the saliva domains is observed, as in this study, and shoulders.

In a previous study using the EORTC QLQ-C30/ QLQ-H&N35 questionnaire, an increase in dry mouth and salivary viscosity with significant impairment in swallowing capacity was observed [ 1 ]. The result is in line with the present study in which dry mouth, sticky saliva, nausea and vomiting were the items with lower scores. Yet, using the same questionnaire in 18 patients, Ohrn *et al*. [ 38 ] found diet and social contact with higher means before and after radiotherapy. Abendstein *et al*. [ 39 ] showed that, after five years of follow-up, symptoms of sense, sexuality, dental problems, mouth openness and dry mouth were aggravated.

Gwede *et al*. [ 40 ] used the FACT-H&N instrument to evaluate changes in quality of life up to one year after the end of radiotherapy treatment for head and neck cancer. After six months of treatment, patients reported pain in the mouth and throat; speech, chewing and swallowing difficulties; dry mouth; thick saliva and frequent cough. Sawada31 in his research using the same instrument found irritation, depression and sadness in 56% of patients. The three major domains affected after the radiotherapy treatment found by Rampling *et al*.41 were the production of saliva, swallowing and taste. In the present study the lowest averages found were in the items emotional well-being and physical well-being. It is noteworthy that, in this study, all patients had already completed the antineoplastic therapy. Kamatchinathan *et al*. [ 42 ] conducted a study in India with 171 patients using the FACT-H&N instrument and showed that most patients experienced low scores for quality of life. Only a few had a satisfactory level and had a lower score for emotional well-being.

Although there is no gold standard instrument, several authors recommend that the ideal instrument should be short, concise, easy to understand, self-applied by patients to reduce health professional interference, have low cost, and have their psychometric validation criteria well established [ 12 ].

The present study found a significant correlation between the three instruments, so regardless of the questionnaire used, the same result in relation to quality of life is found either in the overall evaluation of the patient or in the evaluation of the specific domains of Pain, Appearance, Activity, Swallowing, Chewing, Speech, Taste, Saliva, Humor and Anxiety. Although they were the most used instruments to analyze the quality of life of this specific patient profile [ 34 ] and had been widely validated [ 42 ], no previous study was found that analyzed the correlation between the three instruments.

However, due to the broad characteristic of the quality of life and because it is subject to interferences of factors of different natures, there is still no instrument capable of fulfilling such task to analyze it completely43. Such amplitude and subjection to interference by external factors of all kinds makes it difficult for such an instrument to be conceived. Elements that have great potential to impact people's quality of life, such as individual religious, cultural and historical factors of each subject are not covered by the questionnaires used in this study.

Finally, given the increasing success rate of antineoplastic therapies and the consequent increase in patient survival [ 44 ], and the need to provide comprehensive and humanized care to these patients [ 45 ], it is suggested that the evaluation of QOL in patients with head and neck cancer be incorporated into clinical practice. That will assist health professionals in the decision-making process of treatment, sequelae control, institution of preventive measures and psychological orientation both to the patient and to their families. Given the correlation between the questionnaires and the particularities of each one, the selection of the instrument for future research involving patients with head and neck cancer should take into account the specific aspects that one wishes to evaluate. 

## Conclusion

Considering the studied population and the methodology used in this research, it is concluded that:

- The socio-demographic profile, prevalence of location and histological type of head and neck cancer and the antineoplastic treatment given to the patients is similar to that of previous studies;
- The UW-QOL, EORTC QLQ-C30/QLQ-H&N35 and FACT-H&N questionnaires presented a statistically significant correlation in the assessment of the patient's overall quality of life and in the specific domains Pain, Appearance, Activity, Swallowing, Chewing, Saliva, Humor and Anxiety.
